# Biosimilars Have Arrived: Rituximab

**DOI:** 10.1155/2018/3762864

**Published:** 2018-03-22

**Authors:** Maria Greenwald, John Tesser, K. Lea Sewell

**Affiliations:** ^1^Desert Medical Advances, Palm Desert, CA, USA; ^2^Arizona Arthritis & Rheumatology Associates, Phoenix, AZ, USA; ^3^Biotechnology Clinical Development, Pfizer Inc., Cambridge, MA, USA

## Abstract

A biosimilar is a biologic product that is highly similar to a licensed biologic (“originator”) such that there are no clinically meaningful differences in safety, purity, or potency between the biosimilar and the originator. As patent protection and data exclusivity for the biologic rituximab expire, several potential biosimilars to rituximab are in development, which could soon lead to the availability of numerous rituximab biosimilars. Biosimilars are evaluated using thorough and rigorous analyses of the potential biosimilar versus the originator biological to confirm similar structure, function, and clinical efficacy as well as safety. Approval of a biosimilar is based upon the totality of the evidence demonstrating similarity to the originator. An understanding of the process of the interchangeable designation of a biosimilar is important in the context of patient outcomes. We conducted an analysis of the properties and benefits of rituximab in the treatment of inflammatory diseases, the development and approval of biosimilars, and the potential benefits of rituximab biosimilars. PubMed and ClinicalTrials.gov databases were searched for “biosimilar” and “rituximab” and regulatory and pharmaceutical company web pages were screened regarding biosimilars in development and specific guidelines developed for the approval of biosimilars. The results indicate that, at present, six rituximab biosimilar candidates are undergoing comparative clinical development, and two were recently approved in the European Union. Our analysis indicates rituximab biosimilars are expected to have a continuing role in treating inflammatory conditions such as rheumatoid arthritis.

## 1. Introduction

The development of biologic therapies has transformed the treatment of a number of serious diseases; however, patient access to these life-changing therapies may be limited [1–5]. Patents and other periods of exclusivity on a number of biologics are nearing expiration or have already expired, and regulatory pathways have been established to allow the development and approval of products called “biosimilars” [6].

A biosimilar is a biologic product produced using the same gene, which is highly similar to an approved biologic (originator) product such that there are “no clinically meaningful differences between the biological product and the reference [originator] product in terms of safety, purity, and potency” [7]. In the United States, legislation allowing the development and the framework by which biosimilars are approved (the Biologics Price Competition and Innovation Act of 2009 [BPCI]) is part of the Affordable Care Act and allows development of competition in the biologic market [7]. The objectives of the BPCI are conceptually similar to those of the Hatch-Waxman Act [8, 9] for generic drugs in that BPCI allows the entry of less-expensive biologic medicines and thereby increases competition and availability [10].

The European Medicines Agency (EMA) has approved more than 20 biosimilars, including biosimilars of the monoclonal antibodies (mAbs) infliximab, etanercept, and adalimumab [11]. In addition, 2 biosimilars of rituximab were recently approved by the EMA [11, 12]. The US Food and Drug Administration (FDA) approved the first US biosimilar in 2015 (filgrastim) and the first biosimilar mAb (infliximab) in April 2016, followed by approvals of biosimilars for etanercept in August 2016 and adalimumab in September 2016 [13].

The biologic rituximab is a mAb used broadly in oncology, hematology, rheumatology, nephrology, and other disciplines. The patent for rituximab expired in Europe in 2013 and will expire in the United States in 2018; therefore, biosimilars of rituximab are in development and emerging (e.g., Truxima® and Rixathon®/Riximyo® approved in Europe) [6, 12]. As biosimilars of rituximab become available, it is important for healthcare providers to understand the potential role of rituximab biosimilars in the treatment of inflammatory diseases. To address this need, we review the features and benefits of rituximab particularly in the treatment of inflammatory diseases, the development and approval of biosimilars, and the role and potential advantages of rituximab biosimilars.

## 2. Methods

Searches of PubMed and ClinicalTrials.gov were conducted and regulatory agency and pharmaceutical company web pages were screened. Search terms included “biosimilar” and “rituximab,” which were used to gather information regarding biosimilar development, as well as a review of specific guidelines developed by regulatory agencies for the approval of biosimilars.

## 3. Development and Approval of Biosimilars

The development of a biosimilar is different from the process applied to a new biologic. The manufacturing process for the originator biologic is proprietary; therefore, a pharmaceutical company developing a potential biosimilar must analyze the originator extensively and use reverse engineering to develop a biologic entity with highly similar structure and function [14]. A small-molecule drug can be fully defined structurally and, therefore, a generic equivalent can be reproduced with an identical chemical structure via a defined chemical synthesis. However, biologics are usually larger, complex proteins produced using a biologic process requiring production in living cells that are more difficult to characterize fully [7, 15]. Clinical performance of biologic drugs may be affected by minor posttranslational structural modifications due to the manufacturing process [15]. Thus, the process of developing a potential biosimilar requires substantial knowledge and expertise regarding the development and manufacture of biologics in order to accurately characterize the originator and create a biologic product with similar clinical efficacy and safety as the originator [14].

As biosimilars cannot be considered generic equivalents to the originator, a rigorous nonclinical analysis is conducted to confirm structural and functional similarity to the originator [7, 15, 16]. This level of analysis does not supersede the requirement for demonstration of similar clinical efficacy and safety [7, 15, 16]. With this guidance, several regulatory agencies have developed specific guidelines for the approval of biosimilars [7, 15, 16]. Although there are minor differences among the guidelines, the process generally involves a stepwise approach to ultimately demonstrate similar clinical efficacy and safety versus the originator [7, 15, 16].

### 3.1. Stepwise Development Process

The aim of a similarity assessment is not to reestablish the mechanism of action (MoA) or to demonstrate efficacy and safety compared with placebo, as these studies have already been performed to support the new drug application of the originator [17]. Biosimilars are evaluated by rigorous testing comprising analytical and functional studies; nonclinical assessments of toxicity (and other product characteristics if their evaluation is feasible in* in vivo* studies); and clinical evaluation of pharmacokinetics (PK), efficacy, and safety compared with the originator [7, 15, 16]. The first step is a detailed analysis of primary amino acid sequence and higher order (secondary and tertiary) structure [7, 15, 16]. Numerous additional relevant analytical characterization studies are performed, and* in vitro* functional evaluations are mandatory to confirm that the biosimilar acts on the same target or physiologic process as the originator [7, 15, 16]. Nonclinical animal studies comparing toxicity of the potential biosimilar may be required as part of the stepwise evaluation of a potential biosimilar [18].

After confirming a high degree of physicochemical and functional similarity, clinical trials are designed to confirm the potential biosimilar has similar efficacy and safety, including similar potency, PK, pharmacodynamics (PD), and immunogenicity, to the originator [7, 15, 16]. To date, studies for all approved biosimilars are required to demonstrate PK similarity between the biosimilar and the originator drug. Intensive PK profiles are typically performed in healthy individuals, unless the MoA precludes their participation (e.g., evaluation of a potential rituximab biosimilar, which causes profound B-cell depletion), in which case PK comparison to the originator must be evaluated in patients [19]. Typically, after evaluation of PK or PK/PD, at least one confirmatory clinical study in patients is conducted to evaluate efficacy and safety of the potential biosimilar versus the originator, although this step is determined on a case-by-case and agency-by-agency basis [7, 8, 15, 16].

### 3.2. Regulatory Approval of Biosimilars

Regulatory approval of a potential biosimilar is based on the totality of the evidence from all comparability analyses conducted during evaluation of the potential biosimilar, not just the clinical data [7, 15, 16]. The decision to grant approval is made on a case-by-case and agency-by-agency basis, following the country or other worldwide agency's biosimilar guidelines (e.g., EMA or World Health Organization [WHO]) [14]. The biosimilar guidelines contain an additional key provision, termed “extrapolation,” in which the biosimilar may receive regulatory approval for multiple indications of the originator without being evaluated in clinical trials for each condition [7, 15, 16]. Using the totality of the evidence, it may be possible to extrapolate efficacy and safety data to the other approved indications of the originator, thus reducing or eliminating the need for duplicative clinical studies of the biosimilar in multiple indications [7, 15, 16].

After the approval of a biosimilar, the designation of interchangeability may be granted. Although the EMA and WHO do not provide guidance on interchangeability, the FDA has issued a draft guidance stating that interchangeability designation may be granted for a biosimilar that is administered more than once to an individual when “the risk in terms of safety or diminished efficacy of alternating or switching between use of the biologic product and the reference product is not greater than the risk of using the product without such alternation or switch” [18]. In this situation, “the biologic product may be substituted for the reference product without the intervention of the health care provider who prescribed the reference product” [18]. The requirements for demonstration of interchangeability typically include a switching study comparing steady-state PK between the biosimilar and the originator after two switches between originator and biosimilar [18].

## 4. Rituximab

### 4.1. What Are the Features and Benefits of Rituximab?

Rituximab is a genetically engineered chimeric murine/human monoclonal immunoglobulin G1*κ* antibody directed against the CD20 antigen of B cells [20, 21]. Rituximab is indicated for non-Hodgkin's lymphoma (NHL), chronic lymphocytic leukemia (CLL; in combination with chemotherapy), rheumatoid arthritis (RA; in combination with methotrexate), and granulomatosis with polyangiitis and microscopic polyangiitis (in combination with glucocorticoids) [20, 21]. Rituximab was first approved for the treatment of relapsed/refractory CD20-positive B-cell NHL in 1997 [22]. In 2006, following priority reviews, the FDA approved rituximab for the treatment of RA as well as the addition of two new indications in NHL: first-line treatment of previously untreated patients with follicular NHL in combination with cyclophosphamide/vincristine/prednisolone chemotherapy and the treatment of low-grade NHL in patients with stable disease or who achieve a partial or complete response following first-line treatment with cyclophosphamide/vincristine/prednisolone chemotherapy [22, 23]. As of 2012, the last year for which this data was published, more than 3 million patients worldwide have been treated with rituximab [24].

The pathophysiology of RA is incompletely understood but is thought to involve activation of an autoimmune response involving many key effector cells and inflammatory modulators [20, 25]. B cells contribute through antigen presentation, the production of rheumatoid factor, anticyclic citrullinated peptides, and other autoantibodies and B-cell cytokines [26]. The B-cell role as an activator of T cells in RA includes provision of a second signal while presenting antigen with major histocompatibility complex class II (MHCII) molecules (via higher association of human leukocyte antigen-antigen D-related complex with MHCII molecules). In addition, B cells have a role in production of proinflammatory cytokines that cause and maintain T-cell activation, proliferation, and proinflammatory activities, all of which contribute to a sustained inflammatory response and aggravating joint damage [26, 27]. The mechanism by which rituximab is effective in RA is not fully understood but is believed to be via targeted depletion of mature circulating and tissue-residing B cells [20, 28–31].

### 4.2. Rituximab in RA and Inflammatory Diseases

Rituximab was approved for treatment of RA based on data from three randomized, double-blind, placebo-controlled studies in patients with active RA who had insufficient response to a tumor necrosis factor (TNF) inhibitor and methotrexate [22]. In pivotal trials comparing combination rituximab versus placebo or methotrexate alone, rituximab plus methotrexate resulted in higher percentages of patients who had clinically significant improvements in disease, such as meeting the American College of Rheumatology criteria for improvement; responses according to the European League Against Rheumatism criteria; changes in the disease activity score in 28 joints; and clinically meaningful improvements in fatigue, disability, and health-related quality of life [28–30]. Overall incidence of adverse events was generally similar across these studies, with most adverse events characterized as mild or moderate in severity [28–30]. In 2006, following an FDA priority review, rituximab in combination with methotrexate was approved for treatment of adult patients with moderately to severely active RA who had inadequate response to one or more TNF antagonist therapy [20, 22]. Subsequently, rituximab was approved in 2011 for the treatment of granulomatosis with polyangiitis (in which antineutrophil cytoplasmic antibodies and myeloperoxidase are involved) and microscopic polyangiitis (in combination with glucocorticoids).

With several types of treatments available, making appropriate decisions in clinical practice remains challenging. Therefore, the American College of Rheumatology and European League Against Rheumatism have developed guidelines for the management of RA [40, 41]. These guidelines include biologic therapies if traditional disease-modifying antirheumatic drugs (DMARDs) such as methotrexate provide insufficient response [40, 41]. Recent guidelines have moved rituximab to a first-line biologic in the US and in certain cases in Europe, as more data demonstrating the efficacy, safety, and optimal dosing strategies with rituximab have become available [40–42]. For example, a recent study demonstrated initial treatment with rituximab was noninferior to initial TNF-inhibitor treatment in patients seropositive for RA and naïve to treatment with biologics, although no X-rays were obtained during this head-to-head comparison [43]. This is a change from older guidelines wherein rituximab was a second-line biologic (after failure of a TNF-inhibitor) [40–42]. The change in RA treatment guidelines occurred despite the fact that the labeled indication for rituximab in RA has remained consistent since approval [20, 21]. The recommended use of rituximab has implications for patient access because of regulatory and/or payer demands of strict enforcement of the labeled indication [1].

The change in treatment guidelines to include rituximab as a first-line biologic is particularly relevant to patients with relative or absolute contraindications to TNF inhibitors, such as those with a personal or family history of lymphoma, history of previous active tuberculosis (TB), or latent TB with contraindications to the use of chemoprophylaxis, those living in a TB- or fungal-endemic region, previous history of demyelinating disease, or those with congestive heart disease [40, 41]. Based on the indications for NHL and CLL, rituximab may be the best choice in the treatment of patients with a family history of lymphoma [20, 21, 44, 45]. In addition, some patients develop antibodies to TNF inhibitors, so alternative treatments are needed. For example, rituximab was shown to be effective in treating a case of long-standing, poorly controlled RA in a patient previously exposed to multiple TNF inhibitors and who developed immune-mediated membranoproliferative glomerulonephritis [46]. Similarly, rituximab improved responses in patients who had inadequate response to a TNF-inhibitor as compared with switching to another anti-TNF agent [43, 47, 48]. Rituximab is approved for Wegener's granulomatosis and microscopic polyangiitis [49], which may be approved indications for any rituximab biosimilar, based on extrapolation of data.

### 4.3. The Role and Advantages of a Rituximab Biosimilar

Surveys of rheumatologists have indicated that access to biologic therapies for RA may vary considerably, and clinicians often encounter limitations or barriers to biologics in clinical practice [3, 4]. Similarly, a recent survey of oncologists indicated that many physicians have encountered barriers to accessing rituximab for treatment in patients with NHL or CLL [1]. Restrictions on or disparity in access to biologics, including rituximab, have also been found in regional analyses and assessments of patient access and cost-effectiveness in rare diseases [2, 5]. A number of factors may be involved in the patient's lack of access to rituximab for the treatment of RA, including restrictive treatment guidelines, administrative hurdles, and financial considerations, for example, insurance/public payer coverage for the biologic and the infusion facility, reimbursement, and out-of-pocket cost to the patient [1, 3, 4]. The availability of biosimilars may be expected to reduce barriers to access, increase use, offer patients a more affordable option, lead to further distribution and earlier initiation of biologics in the disease process, and improve patient outcomes [1]. In addition, the complex pathophysiology of RA and different observed responses, including significant segments of patients who do not receive sufficient benefit with traditional DMARDs and/or TNF inhibitors, suggest that rituximab will have a continued role in the treatment armamentarium for RA.

Because inflammatory diseases require long-term use of the prescribed treatment, patients may benefit by having biosimilars of rituximab available. Annual worldwide sales (US$) of originator rituximab in 2014 were approximately $7.5 billion across all indications [50]. The projected global sales of originator rituximab in 2020 are $5.1 billion (a decrease of ~6%) [50]. The decrease in sales projections for originator rituximab is due to the expected increased competition from the availability of biosimilars and not a decrease in overall sales [50].

### 4.4. Rituximab Biosimilars in Development

Truxima (CT-P10), a biosimilar version of rituximab, was approved in South Korea [51] and Europe [12] for the treatment of RA, CLL, and NHL. In addition, Rixathon (L01XC02) was recently approved in Europe for the treatment of NHL, CLL, RA, granulomatosis with polyangiitis, and microscopic polyangiitis. This rituximab biosimilar has also been approved in Europe as Riximyo (L01XC02) under a duplicate marketing authorization for the treatment of NHL, RA, granulomatosis with polyangiitis, and microscopic polyangiitis [11]. Several rituximab biosimilars are in development (Table 1). Many are expected to be approved, which, in turn, should increase patient access to rituximab and thereby increase its use and distribution [1]. The availability of numerous rituximab biosimilars could ultimately achieve the overarching purpose of the BPCI.

Some potential biosimilars are being developed in systems that may reduce production costs or provide other innovations (Table 1). For example, iBIO has produced a potential biosimilar to rituximab in a nontransgenic green plant, which would allow for lower production costs [36]. Mabion S.A. is developing a potential rituximab biosimilar (currently in clinical trials in patients with RA or lymphoma) that will employ a disposable technology, eliminating the product's contact with the production environment and the machinery in the manufacturing chain [52].

Many of the biosimilar clinical trials designed to confirm comparable efficacy and safety to originator rituximab are being conducted in patients with lymphoma. Through the principles of extrapolation of data for use in an indication held by the originator and for which the biosimilar was not directly studied in a comparative clinical trial [7, 15, 16], it is possible that some rituximab biosimilars may receive regulatory approval for RA (or for Wegener's granulomatosis [i.e., granulomatosis with polyangiitis]) without additional clinical trials in each indication, if it is scientifically justified based on the totality of the evidence. However, because RA patients are considered an appropriate population for PK and PK/PD studies of rituximab, it is likely there will be data for a number of potential biosimilars in studies of patients with RA [19]. In addition, some of the potential rituximab biosimilars in treating patients with RA have confirmatory efficacy and safety clinical trials listed in registries (such as ClinicalTrials.gov). Thus, a considerable amount of rigorous data will demonstrate similar structure, function, and efficacy of rituximab biosimilars for regulatory agencies to use for the extrapolation of data to other indications.

## 5. Conclusions

Rituximab has a unique MoA of B-cell depletion that leads to clinical improvements in patients with a variety of inflammatory diseases, including patients with insufficient clinical response while using another biologic therapy, and in patients for whom other biologics are contraindicated. The availability of biosimilars of rituximab should increase patient access to rituximab. Biosimilars are evaluated using thorough and rigorous analyses of the potential biosimilar versus the originator biological to confirm similar structure, function, and clinical efficacy as well as safety. Due to the potential impact on patient outcomes, an understanding of the process of the interchangeable designation of a biosimilar is important. Health care providers should be aware that biosimilars of rituximab will likely have a role in treating inflammatory conditions such as RA for which long-term treatment is necessary.

## Figures and Tables

**Table 1:** Biosimilars of rituximab in development [32].

Potential biosimilar (manufacturer)^a^	Development details
ABP 798 (Amgen)	Comparative efficacy and safety clinical trials in RA and NHL (ongoing) [33]
BX2336 (BioXpress Therapeutics)	In the company pipeline [34]
CT-P10 (Celltrion/Hospira)	Application submitted to EMA in Nov 2015 [35] and approved Feb 2017 [12]
Plant-produced rituximab biosimilar (iBio)	Rituximab produced in nontransgenic plants [36]
MabionCD20 (Mabion)	Comparative efficacy and safety clinical trials in NHL and RA (ongoing) [37]
PF-05280586 (Pfizer)	PK study in RA (completed); comparative efficacy and safety study in follicular lymphoma (ongoing) [19, 38]
GP2013 (Sandoz)	Application submitted to EMA in May 2016 [39]
L01XC02 (Sandoz)	Application approved by EMA in June 2017 [11]

^a^Other biologics in development or approved under regulatory processes without the rigorous evaluation of biosimilarity defined by the EMA, FDA, or WHO guidelines are not included in this table; EMA, European Medicines Agency; FDA, US Food and Drug Administration; NHL, non-Hodgkin's lymphoma; PK, pharmacokinetics; RA, rheumatoid arthritis; WHO, World Health Organization.
